# Sequence and Configuration of a Novel Bispecific Antibody Format Impacts Its Production Using Chinese Hamster Ovary (CHO) Cells

**DOI:** 10.1002/bit.28879

**Published:** 2024-11-25

**Authors:** Hirra Hussain, Angelica M. S. Ozanne, Tulshi Patel, Davide Vito, Mark Ellis, Matthew Hinchliffe, David P. Humphreys, Paul E. Stephens, Bernie Sweeney, James White, Alan J. Dickson, C. Mark Smales

**Affiliations:** ^1^ Manchester Institute of Biotechnology, Faculty of Science and Engineering University of Manchester Manchester UK; ^2^ School of Biosciences, Division of Natural Sciences University of Kent Canterbury UK; ^3^ UCB Pharma UK, Slough Berkshire UK; ^4^ National Institute for Bioprocessing Research and Training (NIBRT) Dublin Ireland

**Keywords:** bispecific antibodies, BYbe, cell line construction, CHO cells, recombinant antibodies

## Abstract

There are a number of new format antibody‐inspired molecules with multiple antigen binding capabilities in development and clinical evaluation. Here, we describe the impact of the sequence and configuration of a unique bispecific antibody format (termed BYbe) using a panel of four BYbe's and the three IgG1s from which they were derived on their production in a Chinese hamster ovary (CHO) cell expression system. Following transfection and selection, one bispecific antibody format yielded fewer mini‐pools in comparison to the other bispecific cell pools. When the top 12 expressing stable mini‐pools of all BYbe configurations and sequences were evaluated, both the dsscFv sequence and antibody chain configuration or placement directly impacted productivity. The cell‐specific productivity (qP, pg/cell/day) was lower in all BYbe cell pools compared to the IgG1 cell lines. However, when the actual molecules/cell/day produced were considered, three of the four bispecific cell pools outproduced the parental IgG1 cell pools. While gene copy number did not correlate to productivity, mRNA analysis showed that for specific BYbe formats there was a strong correlation with productivity. In summary, we describe how bispecific antibody format configuration impacts the cell line construction process and yield of product from CHO cells.

## Graphical abstract

Bispecific BYbe® format sequence and configuration impacts secretory titre. Variable fragment (Fv) sequence and placement can reduce or enhance titre. Vector development strategies that allow tuning and rapid assessment of the ratios of transcripts and polypeptides of different chains in bispecific molecules will facilitate optimisation of vector design for enhanced production of bispecific molecules.

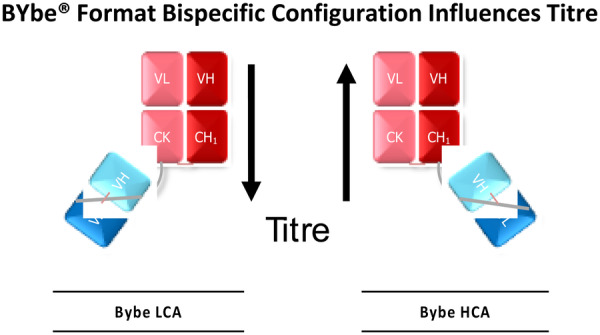

## 1 Introduction

Monoclonal antibodies (mAbs) and their derivatives are a major class of biopharmaceuticals used for the treatment of a range of diseases and conditions (Budge et al. 2020). The engineering of mAbs has led to the development of nonnative, antibody “inspired” molecules and novel formats such as antigen‐binding fragments (Fabs) (Hussain et al. 2021) and bispecific antibodies (Bhatta et al. 2021), a number of which have been approved for therapeutic use (Sandomenico, Sivaccumar, and Ruvo 2020; Spiess, Zhai, and Carter 2015; Thakur, Huang, and Lum 2018). Bispecific antibodies can bind to two different epitopes simultaneously which may be on two different antigens or on the same antigen. Hence, bispecific antibodies enable novel mechanisms of action in comparison to mAbs, which due to their nature, are specific for a single antigen (Husain and Ellerman 2018). Chinese hamster ovary (CHO) cells are the cell line of choice for producing mAbs with 84% of mAbs produced in CHO cells from January 2014 to July 2018 (Walsh 2018).

At the time of writing, seven bispecific antibodies have been approved for therapy, Amivantamab (Rybrevant), Teclistamab (Tecvayli), Mosunetuzumab (Lunsumio), Cadonilimab (AK104 开坦尼), Faricimab (Vabysmo), blinatumomab (Blincyto), emicizumab (Hemlibra) and there are more than 90 bispecific antibodies in development (Brinkmann and Kontermann 2017; Gökbuget et al. 2018; Lillicrap 2017). The first bispecific antigen binding molecule was created in the 1960s by combining two Fabs (Nisonoff and Rivers 1961). Later, hybridoma technology was developed, which enabled a different approach to develop bispecific antibodies of defined specificities (Köhler and Milstein 1975; Suresh, Cuello, and Milstein 1986). Further strategies for the production of bispecifics were later developed in attempts to overcome problems such as the random association of chains and thus multiple products being produced (such as the same two heavy chains [HCs] associating rather than the two different HCs) and to improve scalability (Husain and Ellerman 2018). Indeed, the “knobs‐into‐holes” approach, whereby mutations that are complementary in the CH_3_ domain of each unique HC in the bispecific molecule are made, sought to favor heterodimer formation over homodimer formation of the HC without using chemical conjugation or linkers (Ridgway, Presta, and Carter 1996). The fusion of antibody fragments enabled the development of a “diabody,” a bispecific molecule consisting of the heavy (V_H_) and light (V_L_) chain variable domains of two different antibodies linked on the same polypeptide chain (Holliger, Prospero, and Winter 1993). Since these early approaches, the number of different bispecific antibody formats and approaches to drive correct assembly/heterodimer formation has grown (see e.g., review by Amash et al. 2024 for more detail). To make the production of these bispecific antibodies viable, the correct monomer format needs to be the dominant molecule produced (ideally the only) and at sufficient titer and product quality. Thus, the investigation of different formats of bispecific antibodies with the same antigen binding domains is key to understanding how this impacts yield and facilitates their continued development.

**Figure 1:**
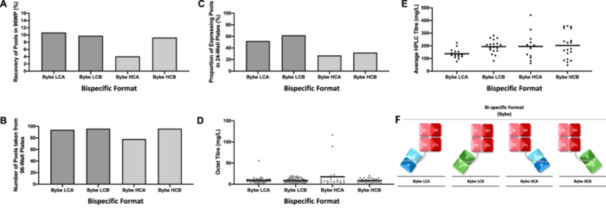
Summary of the cell line development process for the generation of bispecific (BYbe) antibody‐producing mini‐pools. The recovery of mini‐pools post‐transfection is shown as the percentage proportion of wells in 96‐well plates that contained a single colony compared to the total number of wells seeded (A). Further, the number of pools progressed from 96‐well plates to 24‐well plates are shown (up to a maximum of 96 mini‐pools per cell line) (B). From the 24‐well stage, the percentage proportion of mini‐pools that give a detectable level of product above negative controls on an Octet protein G assay are shown (C), as well as the titre readings and mean (solid black bar) for all mini‐pools (D). After progression to the 125 mL shake flask stage, HPLC (Protein G) analysis was carried out on all mini‐pools on supernatant samples collected after 9 days of batch culture and titre measurements and mean titre (*solid black bar*) plotted (E). Schematics show the design of the four bispecific antibody formats (F). Schematics are shown for all formats where each colour (*red, blue and green*) represent the three unique antigen targets.

Others have previously investigated a Fab‐dsFv format (Fab = antigen‐binding fragment, dsFv = variable fragment engineered to contain an inter‐domain disulfide [ds] bond), where the variable light and variable heavy domains of anti‐human serum albumin Fv (variable fragment) were individually linked via peptide linkers to the Fab region constant light and heavy domains (Davé et al. 2016). The anti‐albumin Fv was used to extend the half‐life of the molecule (Davé et al. 2016). Other similar bispecific formats reported are Fab‐dsscFv (disulfide stabilized single‐chain variable fragment), where a single‐chain variable fragment is attached to the Fab (Bhatta and Humphreys 2018). Here, we have investigated the production of the Fab‐dsscFv format and its configuration in CHO cells using four different bispecific antibodies (termed BYbe's). The BYbe sequences were derived from three different IgG1 molecules, termed IgG1 X, Y, and Z. The BYbe antibodies all contain the same antigen‐binding domain (Fab) with the corresponding antigen specificity from hIgG1 X. To this common Fab element, a single‐chain variable fragment (V_H_ and V_L_ domain) of either hIgG1 Y (Antigen A) or hIgG1 Z (Antigen B) was attached to either the light chain C_L_ domain or to the heavy chain C_H_1 domain to generate a BYbe molecule (see Figure 1F). Our findings show the configuration and sequence of the BYbe impact the number of cells that recover posttransfection and the yield of secreted product from CHO cells.

## 2 Materials and Methods

### 2.1 Cell Lines and DNA Constructs

CHO‐DG44 suspension cells (UCB) were grown in commercial DG44 medium (Life Technologies). The appropriate light and heavy chain genes were synthesized and cloned into UCB proprietary double gene vectors. A schematic with more information on the expression vector configuration, including promoters and selection marker, is provided in Supporting Information S1: Schematic 1. For full‐length antibodies, HCs contained the same human IgG1 C_H_1, C_H_2, and C_H_3 domain and the same human kappa C_L_ light chain domain but with V_H_ and V_L_ domains with different antigen specificity termed X, Y, and Z. The BYbe antibodies generated all contained the same heavy chain C_H_1 and light chain C_L_ domain with V_H_ and V_L_ domains with the corresponding antigen specificity from the hIgG1 X sequence. To this common Fab element, for all of the BYbe, the dsscFv (V_H_ and V_L_ domains) of either hIgG1 Y (Antigen A) or hIgG1 Z (Antigen B) was attached to either the light chain C_L_ domain or to the heavy chain C_H_1 domain. Seven molecules in total were generated for analysis, hIgG1 X, hIgG1 Y, hIgG1 Z, BYbe LCA, BYbe LCB, BYbe HCA, and BYbe HCB (see Figure 1F for schematic depiction).

### 2.2 Stable Cell Mini‐Pool Generation

Stable CHO‐DG44 (UCB) mini pools were generated by transfecting linearized DNA encoding the BYbe or IgG1 molecules using the Cell Line Nucleofector V Kit (Lonza) according to the manufacturer's instructions. Stable cell mini‐pool generation was then achieved, as detailed by Hussain et al. (2021). In summary, 1 × 10^7^ cells and 100 µg linearized DNA were used per T‐75 flask which were electroporated in 1 mL of nucleofector solution. At 24 h posttransfection, cells were placed into selective CD‐CHO media (supplemented with glutamine and methotrexate [MTX]) and plated out into 96‐well plates. For each transfection, 20 96‐well plates were plated out at 4000 cells per well. After 14 days, single colonies (termed mini‐pools) were identified using a CloneSelect Imager (Molecular Devices) and transferred to 24‐well plates. The mini‐pools were grown in selective CD‐CHO media for 10 days, and then culture supernatant was sampled for product titer analysis using an Octet QK with Protein G biosensors (FortéBio). The top‐20 titer ranking mini‐pools were progressed eventually to 125 mL Erlenmeyer shake flasks and grown in HyClone ActiSM medium (with glutamine and MTX).

### 2.3 Antibody Titer Analysis by High Performance Liquid Chromatography (HPLC)

Titer analysis to determine antibody expression and concentration in cell culture supernatants was undertaken by HPLC analysis as described in Hussain et al. (2021). Briefly, culture supernatants were harvested from shake flasks by centrifugation and analyzed on an Agilent Technologies 1200 series HPLC system, using a Protein G column and Agilent Chemstation (Rev.B.04.03(16)) software for analysis.

### 2.4 SDS‐PAGE and Western Blot Analysis

The preparation of intracellular and secreted protein samples, SDS‐PAGE and western blot analysis were carried out as described in Hussain et al. (2021). Briefly, proteins were resolved by SDS‐PAGE and transferred onto nitrocellulose membranes that were subsequently blocked in 5% (w/v) milk in phosphate‐buffered saline (PBS) with 0.1% (v/v) Tween‐20 (5% mPBS‐T) before incubation with primary antibodies. Primary antibodies used were an anti‐human C_H_1 domain antibody (generated in‐house by UCB), anti‐human kappa antibody (1:1000, cat no. 9230‐01; Southern Biotech), and anti‐ERK2 (1:1000, cat no. sc‐81459; Santa Cruz Biotechnology) as an intracellular sample loading control. Quantification was undertaken using the Bio‐Rad Image Lab software (Version 6.0.1). All statistical analysis was performed using GraphPad Prism (Version 8.4.0).

### 2.5 RNA Extractions, RT‐qPCR, and Genomic DNA (gDNA) Extractions

RNA extractions, RT‐qPCR and gDNA extractions were again carried out as described by Hussain et al. (2021). Briefly, total RNA was extracted from frozen cell pellets using the commercially available mirVana miRNA Isolation Kit with phenol and eluted in DNase‐free water. For RT‐qPCR, the Power SYBR Green RNA‐to‐CT 1‐Step Kit (Thermo Scientific) was used with 80 ng of RNA for each reaction. Transcript numbers were quantified using a standard curve. gDNA was extracted from cell pellets using the commercially available PureLink Genomic DNA Kit (Invitrogen). The copy number was quantified using a plasmid standard curve.

## 3 Results and Discussion

### 3.1 Recovery From Transfection, Selection and Subsequent Productivity of BYbe Cell Mini‐Pools Is Impacted by the Sequence and Architecture of the BYbe Format

We expressed four different BYbe molecules in a CHO cell expression system. As outlined above, the BYbe molecules all had a common Fab domain from the hIgG1 X sequence. To this, a single‐chain variable fragment (dsscFv) was added containing the V_H_ and V_L_ domains of either hIgG1 Y (Antigen A) or hIgG1 Z (Antigen B). The dsscFv was attached to either the light chain C_L_ domain or to the heavy chain C_H_1 domain, to produce BYbe LCA, BYbe LCB, BYbe HCA and BYbe HCB, respectively (Figure 1F). We examined how the different BYbe molecules impacted the cell pool recovery post‐transfection and the progression of the mini‐pools generated through the cell line development process (Figure 1A–C).

Posttransfection cells were plated out into 96‐well plates, and after 2–3 weeks, the number of single colonies (mini‐pools) that recovered as a percentage of those that were plated out was determined (Figure 1A). The percentage recovery of BYbe HCA (4.1%) was less than half that observed for BYbe LCA, BYbe LCB, and BYbe HCB (9.3%–10.7%) (Figure 1A). The fact that fewer BYbe HCA transfectants recovered suggests that BYbe HCA imposes a larger stress on the cells than the other BYbe sequences. Thus, differences were observed in the recovery, depending on the sequence of the dsscFv domain (Antigen A vs. B) and its placement on the BYbe molecule (light chain vs. heavy chain). We have previously reported that the percentage recovery of the full‐length IgG1 molecules from which the BYbe's were derived was between 2% and 4% (Hussain et al. 2021). As the BYbe cell pools recovered as well as or better than the IgG1 molecules, this suggests the burden on the cell recovering from transfection of the BYbe molecules is certainly no more, and possibly less, than from an IgG1 molecule. We suggest that the cell pool data is likely to reflect how these molecules behave generally in pools and stable CHO cell lines. Others have previously reported that findings in CHO cell pools can reflect those in clonal cell lines (Budge et al. 2020; Josephides et al. 2020; Muralidharan‐Chari et al. 2021), which provides confidence that the findings in pools broadly reflect that of clonal cell lines. We acknowledge that individual clonal cell lines can display vastly diverse characteristics. Further, our “pool” generation process (see Section 2) involves selecting a single colony for each pool using a CloneSelect Imager giving further confidence that each pool is likely to reflect clonal cell line characteristics.

The poorer recovery of BYbe HCA impacted the number of mini‐pools available to be taken forward from the 96‐well plate stage for this molecule. Hence, only 78 BYbe HCA mini‐pools were taken forward into 24‐well plates in comparison to the other BYbe's, where 96 were taken forward (Figure 1B). A 9‐day batch overgrow (a closed culture system where all nutrients are provided at the start of culture and no additional feeding is provided during the culture which is run until termination, during or after the death phase, typically when the culture viability has decreased towards 0%) was then carried out in 24‐well plates to assess the product titer in the culture medium on an Octet instrument (Protein G). The proportion of expressing pools was higher in BYbe LCA (52%) and BYbe LCB (62%) in comparison to BYbe HCA (27%) and BYbe HCB (32%) (Figure 1C), however, the mean titer measurements of the expressing mini‐pools were similar (Figure 1D). The top 20 titer mini‐pool producers were then progressed to 125 mL Erlenmeyer shake flasks, passaged 3–4 times and then a 9‐day batch overgrow was carried out to assess the titer by HPLC (Protein G). The average titer of the 20 mini‐pools assessed at the 125 mL shake flask scale was much lower in BYbe's LCA (137 mg/L) than that from BYbe LCB and BYbe HCB (194 and 202 mg/L, respectively) (Figure 1E). The titer data was also examined to determine if the rank of pool expression was preserved from the 24‐well plate to the shake flask stage, which might facilitate the identification of high producers early in the cell line development process. However, the titer ranks differed at each stage, confirming the necessity of screening throughout the cell line development process to identify and isolate the highest‐producing BYbe mini‐pools. Collectively, the recovery data suggests that the dsscFv sequence and site of attachment to the core Fab impacted the number of mini‐pools that survived posttransfection in 96‐well plates, the number of mini‐pools that expressed product at the 24‐well plate stage and the average titers at the shake flask stage.

### 3.2 Growth Profile Characteristics Are Similar Between Different BYbe Molecule Expressing CHO Cell Mini‐Pools

**Figure 2:**
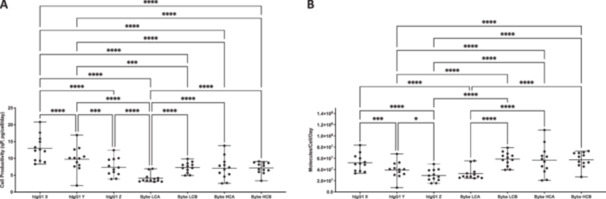
Cell specific productivity of full‐length antibody (hIgG1) and bispecific antibody (BYbe) formats over a 9‐day batch culture. The cell specific productivity (*qP, pg/cell/day*) was compared for the top 12 full‐length hIgG1 and BYbe mini‐pools. Titre was measured on days 3, 6 and 9 of culture using HPLC (mg/L) from culture supernatant samples. The cell specific productivity (*qP, pg/cell/day*) was calculated using the titre and corresponding integral of viable cell concentration (× 10^6^ cells/mL/day) on the same days of culture (A). From the qP, the molar ratio was calculated taking into account the molecular weight for the hIgG1 (150 kDa) and BYbe molecules (75 kDa) (B). The mean is reflected in each plot as a horizontal solid black bar and the range (minimum and maximum values) highlighted with vertical black bars. Statistical analysis by one‐way ANOVA is shown (detailed in Supporting Information S2: Table 1), where *p* values of < 0.05 (*), < 0.01 (**), < 0.001 (***), and < 0.0001 (****) were deemed significantly different. Those comparisons that were significant are shown.

**Figure 3:**
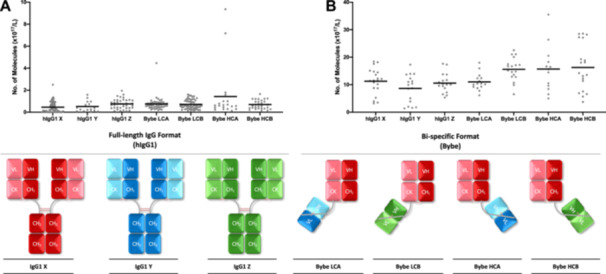
Comparison between the molar concentration of IgG and BYbe in mini‐pools. The molar concentrations were calculated for the Protein G titre measurements at the 24‐well stage (A) and HPLC titre measurements at the 125 ml shake flask stage (B). Comparisons were made across the originator IgG molecules (antigens X, Y, and Z) and the novel format bispecific (BYbe) antibodies shown in the schematics above where each colour (*red, blue, and green*) represent the three unique antigen targets. The molar concentrations were calculated taking into account the molecular weight for an hIgG1 (150 kDa) and BYbe (75 kDa) molecule. The total number of molecules for the number of moles was calculated using Avogadro's number (1 mole = 6.022 × 10^23^ molecules). The mean for each data set is reflected in each plot as a solid black bar.

**Figure 4:**
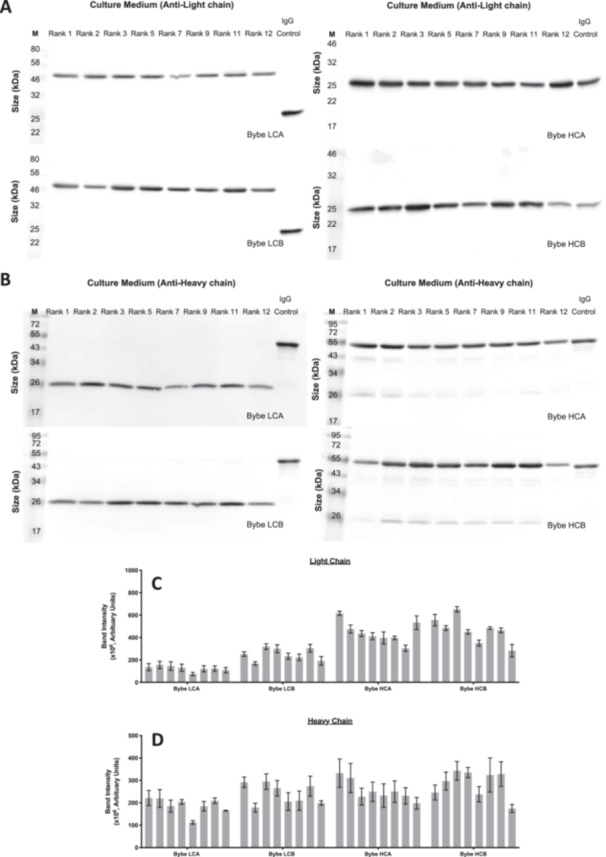
Analysis of secreted light and heavy chain in cell culture medium samples for all BYbe mini‐pools. Culture medium was harvested on day 6 of culture (end of exponential growth phase) for secreted protein analysis. Samples were analysed by western blot under reducing conditions (with 1.8% (v/v) β‐mercaptoethanol). Western blot images and quantitation are shown for light chain (A, C) and heavy chain (B, D) detection for 8 out of 12 mini‐pools selected for each cell line with a range in qP. Data is shown in rank order (decreasing qP). A commercial IgG was used as a positive control (IgG control). Data is representative of at least three replicates and error bars show the mean ± standard deviation (n ≥ 3).

From the initial shake flask data, the top 12 mini‐pools based on the HPLC titer data were selected for a 9‐day batch overgrow for further investigation. The growth profiles of all the mini‐pools were found to be similar (Supporting Information S1: Figure 1). To further examine the growth characteristics, the specific growth rate in the exponential phase and doubling time were calculated which revealed that BYbe LCB had both a significantly average lower specific growth rate (*µ*, 0.019 h^−1^) and a higher doubling time (37.88 h) than the other BYbe cell lines (ranging from *µ* = 0.027–0.030 h^−1^ and 23.13–26.57 h) (Supporting Information S1: Figure 2). However, no statistically significant correlation was observed between the specific growth rates and the cell‐specific productivity (qP) for any of the BYbe's (Supporting Information S1: Figure 3). The average cell diameter of all the mini‐pools decreased over time, with no significant differences observed between the different BYbe's (Supporting Information S1: Figure 4).

### 3.3 Analysis of Product Titer on the Basis of Cell Specific Productivity Reveals Differences Between BYbe and That CHO Cells Are Just as Efficient at Making BYbe as Full‐Length IgGs

**Figure 5:**
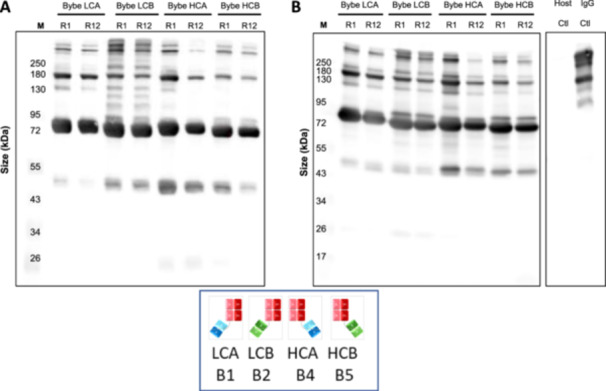
Analysis of secreted light and heavy chain in culture medium samples under nonreducing conditions. Culture medium was harvested on day 6 of culture (end of exponential growth phase) for secreted protein analysis. Samples were analysed by western blot under non‐reducing conditions. Images are shown for the light chain (A) and heavy chain (B). Data is shown for top (R1) and bottom (R12) ranked mini‐pools selected for each molecule. Culture medium from a non‐transfected host cell line culture (Host Ctl) was used as a negative control. A commercial IgG was used as a positive control (IgG control). Data shown is representative of at least three replicates.

The qP range, between the highest and lowest producers of the top 12 BYbe HCA mini‐pools, was greater (5.36‐fold difference) than for the other BYbe mini‐pools (2.00–2.73‐fold difference) (Figure 2A and Supporting Information S1: Figure 5). In addition, the highest BYbe HCA producer had a qP nearly twofold greater than the highest BYbe LCA producer (13.78 vs. 6.91 pg/cell/day). In contrast, the highest producers of BYbe LCB and BYbe HCB had similar cell productivity (9.85 and 9.09 pg/cell/day, respectively). The qP of the BYbe was also compared to the IgG1 molecules that they were derived from and shows that the mean qP was significantly higher (*p* < 0.0001, Supporting Information S2: Table 1) in the IgG1 X molecule compared to all the BYbe. In addition, the mean qP was significantly higher in IgG1 Y in comparison to BYbe LCA and BYbe HCA (*p* < 0.0001, Supporting Information S2: Table 1) but there were no significant differences between the mean qP of IgG1 Z and BYbe LCB (*p* > 0.9999) and BYbe HCB (*p* = 0.9997, Supporting Information S2: Table 1).

Protein G secretory titer data for samples from the 24‐well plates (Figure 1D) and the HPLC titer data from the 125 mL Erlenmeyer flasks (Figure 1E) was also converted to the number of molecules produced per cell as opposed to the mass of product produced per cell (Figure 2B). As the BYbe molecules all have a similar molecular mass, this did not change the trends observed within the BYbe data. When the number of molecules produced per volume titer was calculated (molecules/L as opposed to g/L) for the full‐length IgG and BYbe's at the 24‐well plate and 125 mL shake flask scale, the BYbe's generally outperformed the equivalent IgGs (Figure 3). Similarly, conversion of the cell‐specific productivity (pg/cell/day to molecules/cell/day) also allowed a better comparison between the number of BYbe and IgG1 molecules produced and consideration of the mass difference between the formats. When the number of molecules produced were considered the mean BYbe molecules/cell/day was very similar to IgG1 X and only significantly lower for BYbe LCA (*p* < 0.0001, Figure 2B and Supporting Information S2: Table 1). In addition, the top‐producing mini‐pool of BYbe HCA produced more molecules/cell/day than the equivalent IgG1 X producer (Figure 2B). Thus, it is evident that the measurement used to assess productivity can lead to differing conclusions, regarding the difficulty of expressing the BYbe molecules in comparison to the IgG1 molecules from which they are derived.

### 3.4 All BYbe Configurations Are Secreted With No Evidence of Intracellular Accumulation

**Figure 6:**
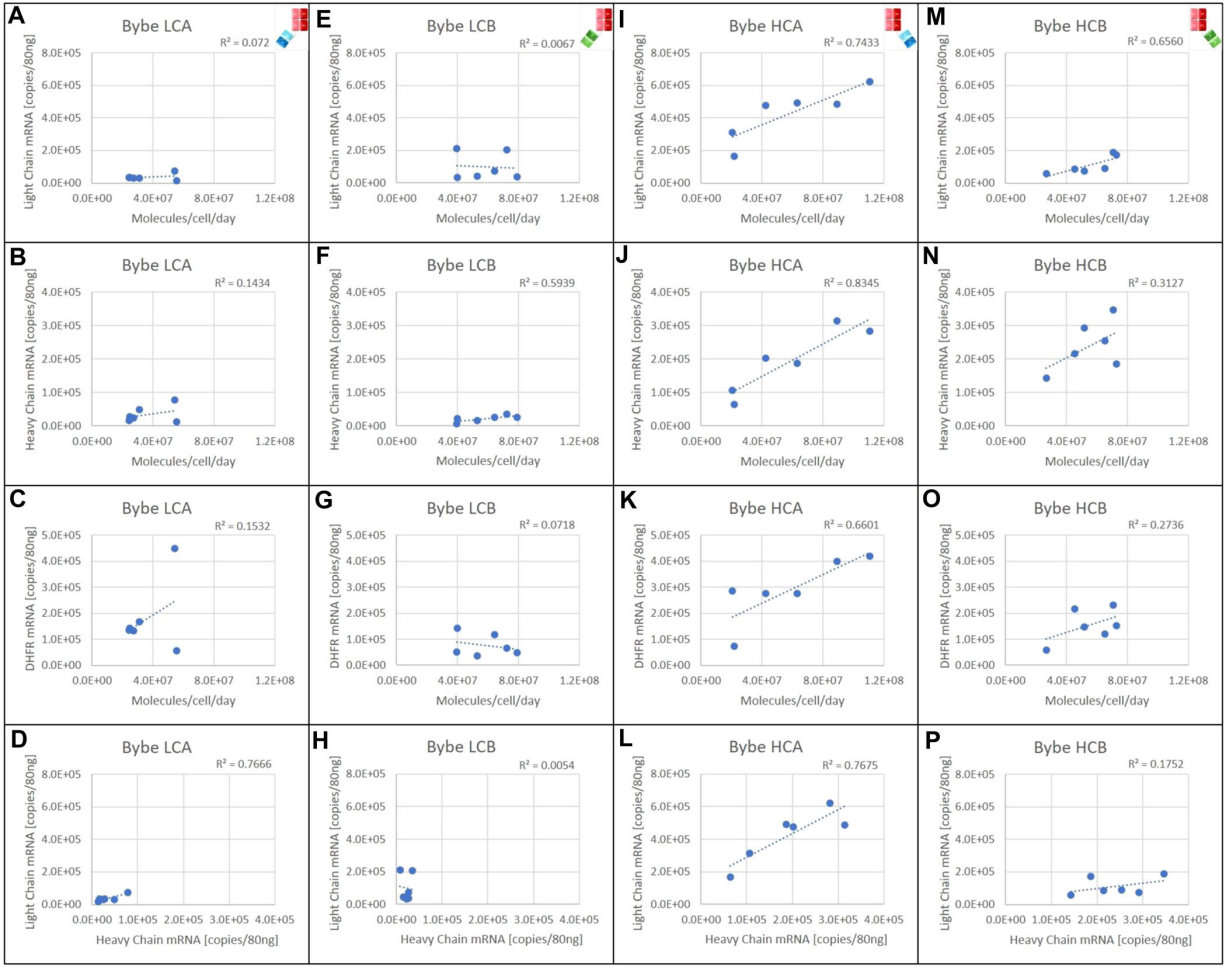
Messenger RNA analysis of all BYbe mini‐pools. Cell pellets were collected on day 6 of culture for mRNA analysis. Light chain, heavy chain and DHFR mRNA copies were quantified for 6 of the mini pools. The mRNA copies of light chain, heavy chain and DHFR were plotted against the molecules/cell/day for BYbe LCA (A, B, and C respectively), BYbe LCB (E, F, and G respectively), BYbe HCA (I, J, and K respectively) and BYbe HCB hIgG1 (M, N, and O respectively). The light chain and heavy chain mRNA copies were also plotted against each other for BYbeLCA (D), BYbe LCB (H), BYbe HCA (L), and BYbe HCB (P).

A subset of 8 mini‐pools, which spanned the range of productivity of each BYbe, were selected for the assessment of secreted and intracellular BYbe protein on Day 6. As all the BYbe's share the same common element of the IgG1 X Fab region detailed previously, this enabled all HCs to be detected with an anti‐human C_H_1 domain antibody (generated by UCB) and the light chains to be detected with an anti‐human kappa antibody. Distinct bands of the expected sizes were observed for both the heavy and light chains, with no or minimal detection of degraded protein observed in culture medium samples (Figure 4). Interestingly, densitometry analysis of the heavy and light chain protein in the culture medium for BYbe LCB and HCB, which have very similar productivities, suggests that when the dsscFv is attached to the light chain as in BYbe LCB, this results in an overall decrease of light chain protein as shown by reduced western blot. However, when the dsscFv is attached to the HC, there is no effect on HC protein amounts in comparison to the other BYbe format (Figure 4). The corresponding intracellular blots show that little heavy and light chain is retained within the cell (Supporting Information S1: Figure 6).

The highest and lowest ranked producers were then selected for nonreducing western blot analysis to examine the assembly of the BYbe molecules. The secreted BYbe predominantly exist in their monomeric forms (Supporting Information S1: Figure 7), and there was little difference between the observed dimers/multimers and free light/heavy chain, in the highest and lowest ranked mini‐pools (Figure 5). In comparison to the IgG1 molecule, there were more dimers/multimers observed which suggests that the BYbe molecules have a higher propensity to form aggregates. Intracellularly, the predominant species observed is the monomeric form (Supporting Information S1: Figure 8), and side‐by‐side comparison with the culture medium protein samples shows that this is secreted out of the cell (Supporting Information S1: Figure 9).

### 3.5 gDNA Copy Number Does Not Correlate With Productivity of BYbe's but Messenger RNA (mRNA) Copy Number Does Correlate With Particular Format Configurations

A subset of 6 mini‐pools, which spanned the range of productivity of each BYbe, was selected for the assessment of gDNA (BYbe's gene copy numbers) and mRNA on Day 6. gDNA analysis showed that there was no correlation between the light or heavy chain copies/cell and the molecules/cell/day for any of the BYbe's (Supporting Information S1: Figure 10). Other studies have also shown that gene copy number does not correlate with productivity (Barnes and Dickson 2006; Lattenmayer et al. 2007), and this suggests that the gDNA copy number is not limiting the production of the BYbe molecules. Additionally, no correlation was seen between the gDNA light or heavy chain copies/cell and the corresponding light or heavy chain mRNA transcript number, nor the heavy chain to light chain mRNA ratio and molecules/cell/day (Supporting Information S1: Figure 11). As the DNA has not been targeted to a specific site but integrates randomly into the genome of the cell, site‐specific differences in the transcription rate can potentially cause differences in gene expression (Barnes, Bentley, and Dickson 2003; Lai, Yang, and Ng 2013).

For BYbe LCA, there was a strong correlation between the light and heavy chain mRNA copies (*R* ^2^ = 0.7666), but no correlation was observed between mRNA transcript numbers and the number of molecules/cell/day (Figure 6A,B,D). This suggests for BYbe LCA, the amount of mRNA transcript was not a limiting factor and that the bottleneck may be in posttranscriptional processing, as reported for other recombinant molecules (Hussain et al. 2017; Mead et al. 2015; O'Callaghan et al. 2010). BYbe HCA also had a strong correlation of light and heavy chain mRNA transcripts (*R* ^2^ = 0.7675), but in contrast to BYbe LCA, both the light and heavy chain mRNA transcript numbers had a strong correlation to the molecules/cell/day (*R* ^2^ = 0.7433 and *R* ^2^ = 0.8345, respectively) (Figure 6I,J,L). This suggests for BYbe HCA, the amount of mRNA transcript is a determining factor for BYbe HCA production, as reported for other recombinant targets (Godfrey et al. 2017; Mason et al. 2012; Mead et al. 2009; Pekle et al. 2019). One approach to further increase BYbe HCA production would, therefore, be to investigate approaches that give higher light and heavy chain transcript amounts.

For BYbe LCB and BYbe HCB, no correlation was observed between light and heavy chain mRNA transcripts (Figure 6H,P). Further, BYbe LCB showed no relationship to the light chain mRNA transcripts and the molecules/cell/day but there was a weak correlation with the HC mRNA transcripts and the molecules/cell/day. The opposite was true for BYbe HCB (Figure 6). These results highlight the need for BYbe's to be examined in each configuration, that is, BYbe LCA versus BYbe HCA, to investigate if the mRNA correlates with productivity and thus could be used as a screening tool during the cell line development process to select high producers and also to investigate any bottlenecks which may limit the productivity.

## 4 Summary

This study describes the impact of the sequence and configuration of four different bispecific antibodies on the cell line construction process and the ability of CHO cells to synthesize these products. During cell line construction, differences were observed in the recovery posttransfection, depending on the sequence of the dsscFv domain (Antigen A vs. B) and the placement or configuration on the BYbe molecule (light chain vs. heavy chain). The placement of the scFv domain also impacted the number of expressing mini‐pools that emerged from the cell pool construction process. Later in the cell pool construction process, when the final top 12 titer mini‐pools were identified, it was evident that both the dsscFv domain sequence and placement directly impacted cell productivity. We have also shown that while the mean qP was lower in the final BYbe cell mini‐pools in comparison to the IgG1 cell pools, if productivity is alternatively assessed as the mean molecules/cell/day, then three of the four BYbe cell pools outproduced the corresponding IgG1 cell pools. This suggests that BYbe molecules may generally not be as “difficult‐to‐express” in comparison to IgG1 molecules and highlights the importance of studies to assess the impact of the placement of the dsscFv domain, that is, light chain versus heavy chain, on productivity.

Nevertheless, it is generally accepted that the large number of potential bispecific antibody formats and their increased complexity compared to mAbs means they are more difficult to express (Peltret et al. 2024). Our molecular analysis highlights the need for BYbe's to be examined in each format, and that mRNA correlation with productivity might be an approach to be used as a screening tool during the cell line development process to select high producers and also to investigate any bottlenecks which may limit productivity. Further, the relationship between transcript amounts and BYbe productivity reported here suggests that the tuning of vector design to “optimise” transcript amounts, and hence the ratio of the different chain transcripts and polypeptides, is likely to be an aspect that can be further developed to enhance the productivity of bispecific molecules generally. Indeed, in support of this, others have reported that vector design is an important consideration in the manufacturing of bispecific molecules (Peltret et al. 2024), and that vector design and heterochain ratios influence the titer and amount of correctly assembled bispecific molecule products (Ong et al. 2022; Gong and Wu 2023), and that tuning transcript amounts through alternative splicing to moderate subunit ratios can be achieved for a bispecific molecule (Aebischer‐Gumy et al. 2024). We, therefore, suggest that vector development strategies that allow the tuning and rapid assessment of the ratios of transcripts and polypeptides of the different chains in bispecific molecules will facilitate the optimization of vector design for enhanced production of bispecific molecules from CHO cell expression systems.

## Author Contributions

A.J.D., C.M.S., P.E.S., and B.S. conceived the study. A.J.D., C.M.S., M.E., M.H., D.P.H., P.E.S., B.S., and J.W. supervised the study. H.H., T.P., A.M.S.O., and D.V. designed and completed the experiments. H.H. and A.M.S.O. drafted the manuscript with the support of A.J.D. and C.M.S. All authors reviewed and approved the manuscript before submission.

## Conflicts of Interest

Mark Ellis, Matthew Hinchliffe, David P. Humphreys, and James White are employees of UCB Pharma. Paul E. Stephens and Bernie Sweeney are former employees of UCB Pharma. The other authors declare no conflicts of interest.

## Supporting information

Supporting information.

Supporting information.

## Data Availability

The data that support the findings of this study are available from the corresponding author upon reasonable request.
