# Supplementary material for: Sequence and Configuration of a Novel Bispecific Antibody Format Impacts Its Production Using Chinese Hamster Ovary (CHO) Cells
Source: Biotechnol Bioeng. 2024 Nov 25;122(2):435–44. doi: 10.1002/bit.28879 (PMC11718431; doi:10.1002/bit.28879)
Supplement: Supplementary file 1 — Supporting information. [file BIT-122-435-s001.pptx]

## Slide 1
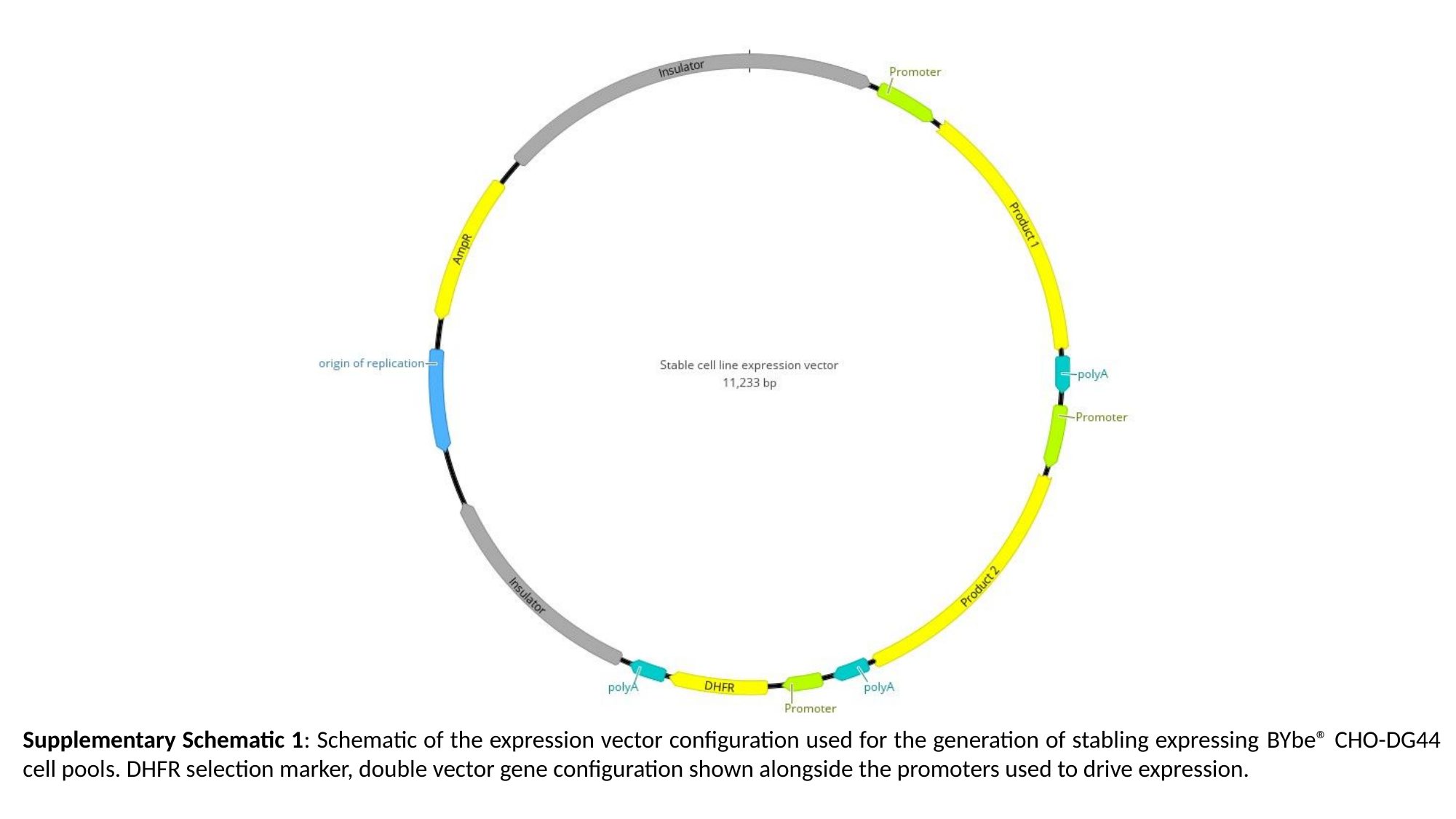

Supplementary Schematic 1: Schematic of the expression vector configuration used for the generation of stabling expressing BYbe® CHO-DG44 cell pools. DHFR selection marker, double vector gene configuration shown alongside the promoters used to drive expression.

## Slide 2
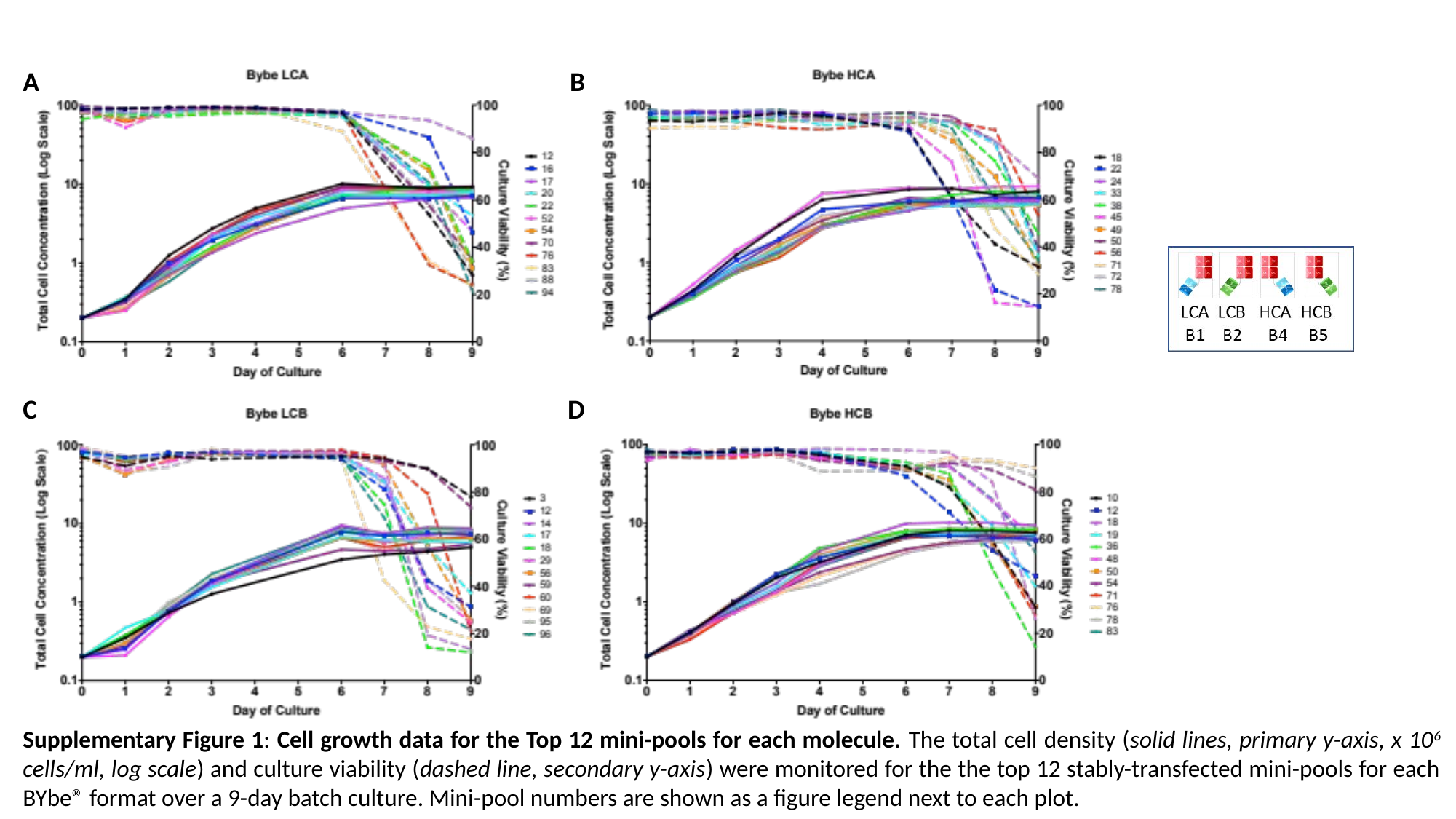

A B
C D
Supplementary Figure 1: Cell growth data for the Top 12 mini-pools for each molecule. The total cell density (solid lines, primary y-axis, x 106 cells/ml, log scale) and culture viability (dashed line, secondary y-axis) were monitored for the the top 12 stably-transfected mini-pools for each BYbe® format over a 9-day batch culture. Mini-pool numbers are shown as a figure legend next to each plot.

## Slide 3
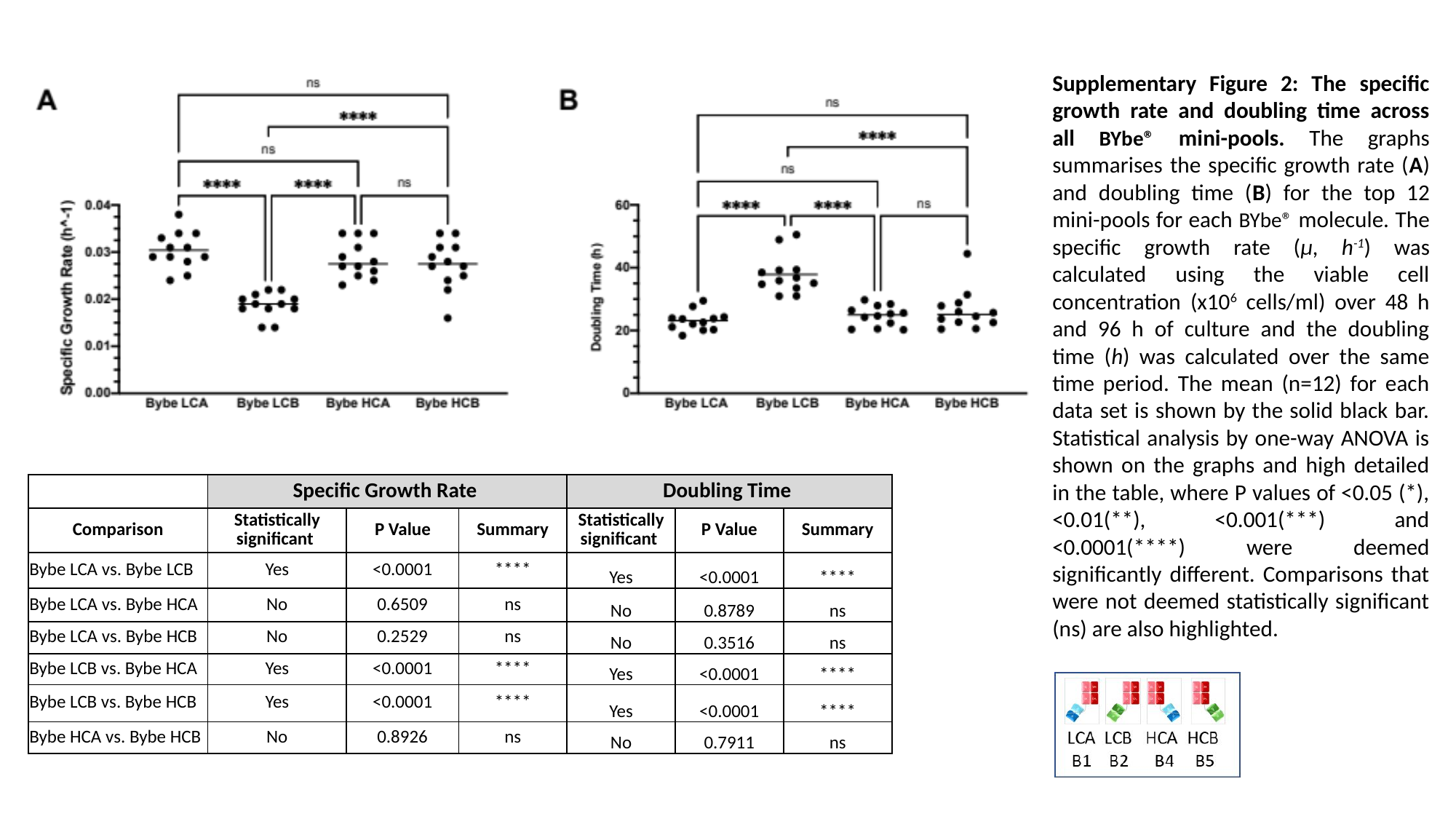

Supplementary Figure 2: The specific growth rate and doubling time across all BYbe® mini-pools. The graphs summarises the specific growth rate (A) and doubling time (B) for the top 12 mini-pools for each BYbe® molecule. The specific growth rate (µ, h-1) was calculated using the viable cell concentration (x106 cells/ml) over 48 h and 96 h of culture and the doubling time (h) was calculated over the same time period. The mean (n=12) for each data set is shown by the solid black bar. Statistical analysis by one-way ANOVA is shown on the graphs and high detailed in the table, where P values of <0.05 (*), <0.01(**), <0.001(***) and <0.0001(****) were deemed significantly different. Comparisons that were not deemed statistically significant (ns) are also highlighted.
| | Specific Growth Rate | | | Doubling Time | | |
| --- | --- | --- | --- | --- | --- | --- |
| Comparison | Statistically significant | P Value | Summary | Statistically significant | P Value | Summary |
| Bybe LCA vs. Bybe LCB | Yes | <0.0001 | \*\*\*\* | Yes | <0.0001 | \*\*\*\* |
| Bybe LCA vs. Bybe HCA | No | 0.6509 | ns | No | 0.8789 | ns |
| Bybe LCA vs. Bybe HCB | No | 0.2529 | ns | No | 0.3516 | ns |
| Bybe LCB vs. Bybe HCA | Yes | <0.0001 | \*\*\*\* | Yes | <0.0001 | \*\*\*\* |
| Bybe LCB vs. Bybe HCB | Yes | <0.0001 | \*\*\*\* | Yes | <0.0001 | \*\*\*\* |
| Bybe HCA vs. Bybe HCB | No | 0.8926 | ns | No | 0.7911 | ns |

## Slide 4
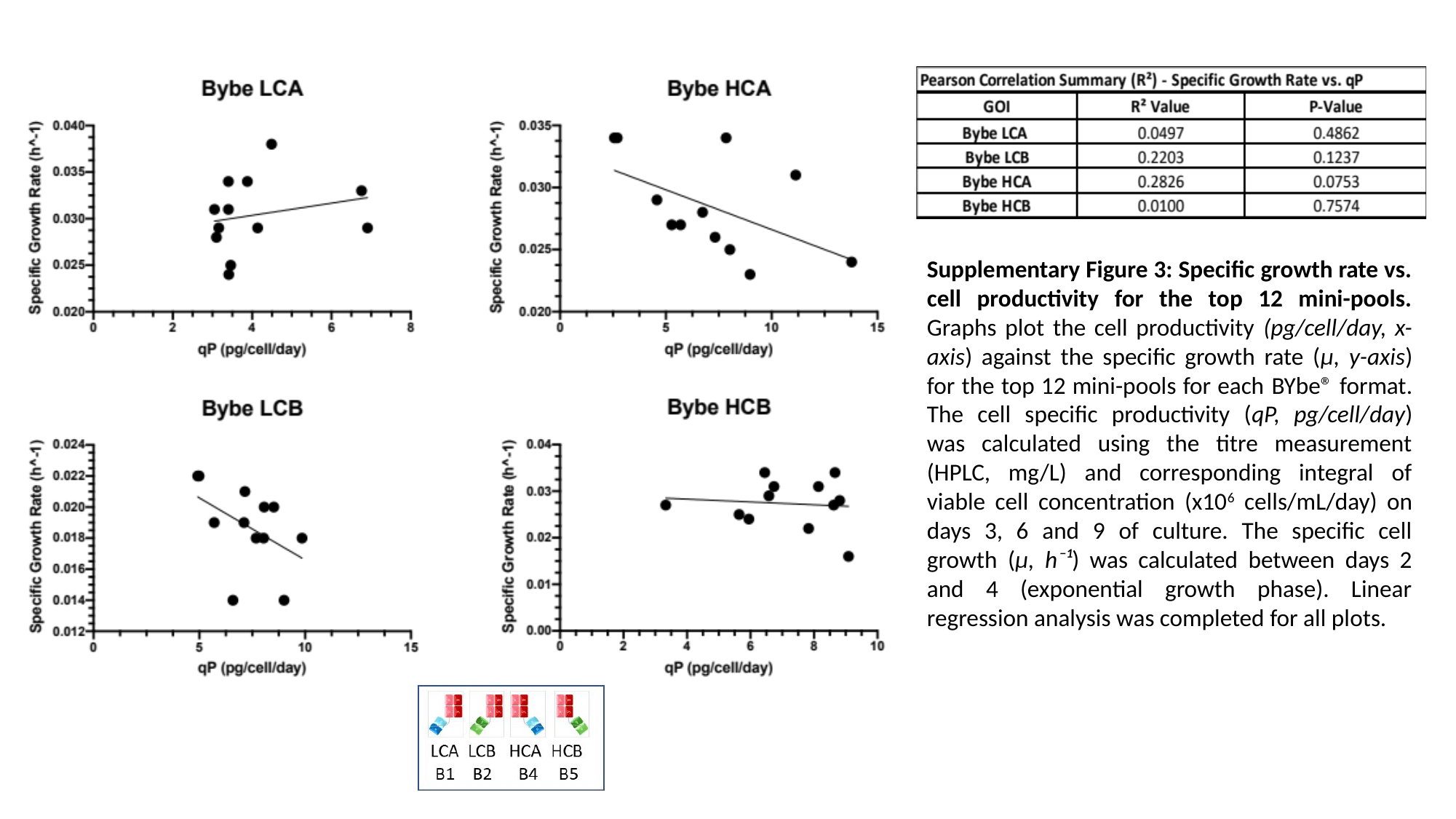

Supplementary Figure 3: Specific growth rate vs. cell productivity for the top 12 mini-pools. Graphs plot the cell productivity (pg/cell/day, x-axis) against the specific growth rate (µ, y-axis) for the top 12 mini-pools for each BYbe® format. The cell specific productivity (qP, pg/cell/day) was calculated using the titre measurement (HPLC, mg/L) and corresponding integral of viable cell concentration (x106 cells/mL/day) on days 3, 6 and 9 of culture. The specific cell growth (µ, h⁻¹) was calculated between days 2 and 4 (exponential growth phase). Linear regression analysis was completed for all plots.

## Slide 5
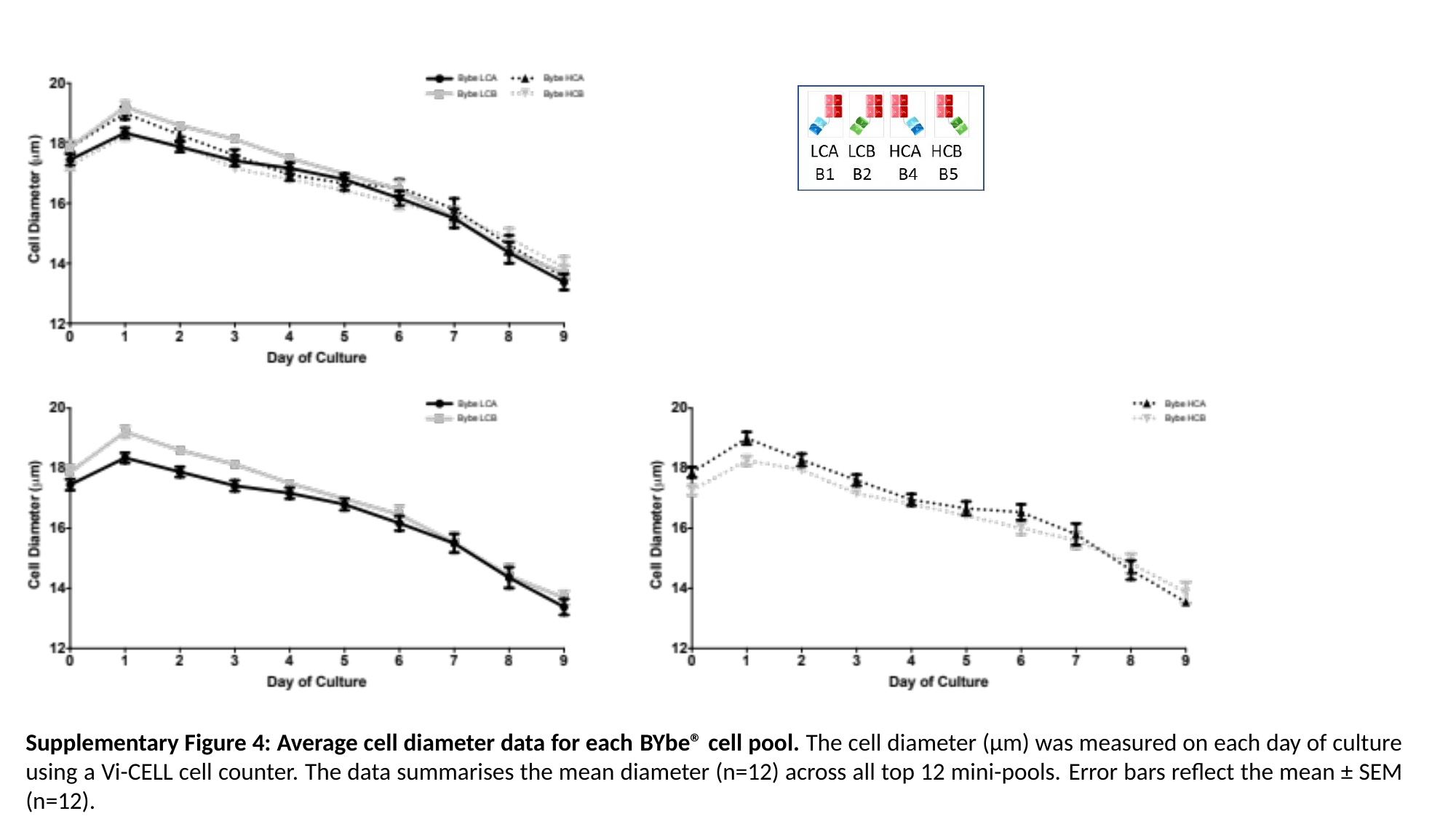

Supplementary Figure 4: Average cell diameter data for each BYbe® cell pool. The cell diameter (µm) was measured on each day of culture using a Vi-CELL cell counter. The data summarises the mean diameter (n=12) across all top 12 mini-pools. Error bars reflect the mean ± SEM (n=12).

## Slide 6
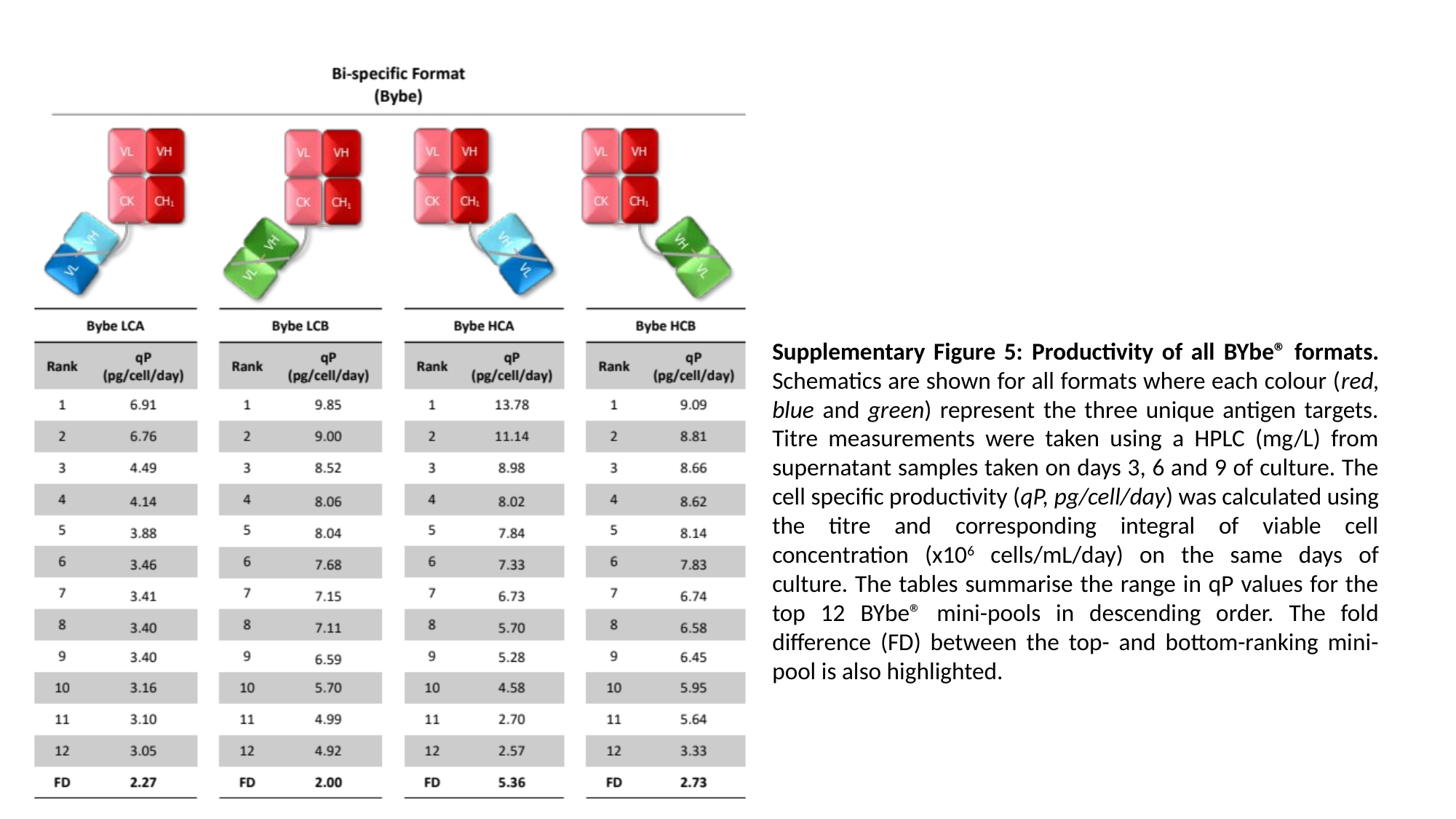

Supplementary Figure 5: Productivity of all BYbe® formats. Schematics are shown for all formats where each colour (red, blue and green) represent the three unique antigen targets. Titre measurements were taken using a HPLC (mg/L) from supernatant samples taken on days 3, 6 and 9 of culture. The cell specific productivity (qP, pg/cell/day) was calculated using the titre and corresponding integral of viable cell concentration (x106 cells/mL/day) on the same days of culture. The tables summarise the range in qP values for the top 12 BYbe® mini-pools in descending order. The fold difference (FD) between the top- and bottom-ranking mini-pool is also highlighted.

## Slide 7
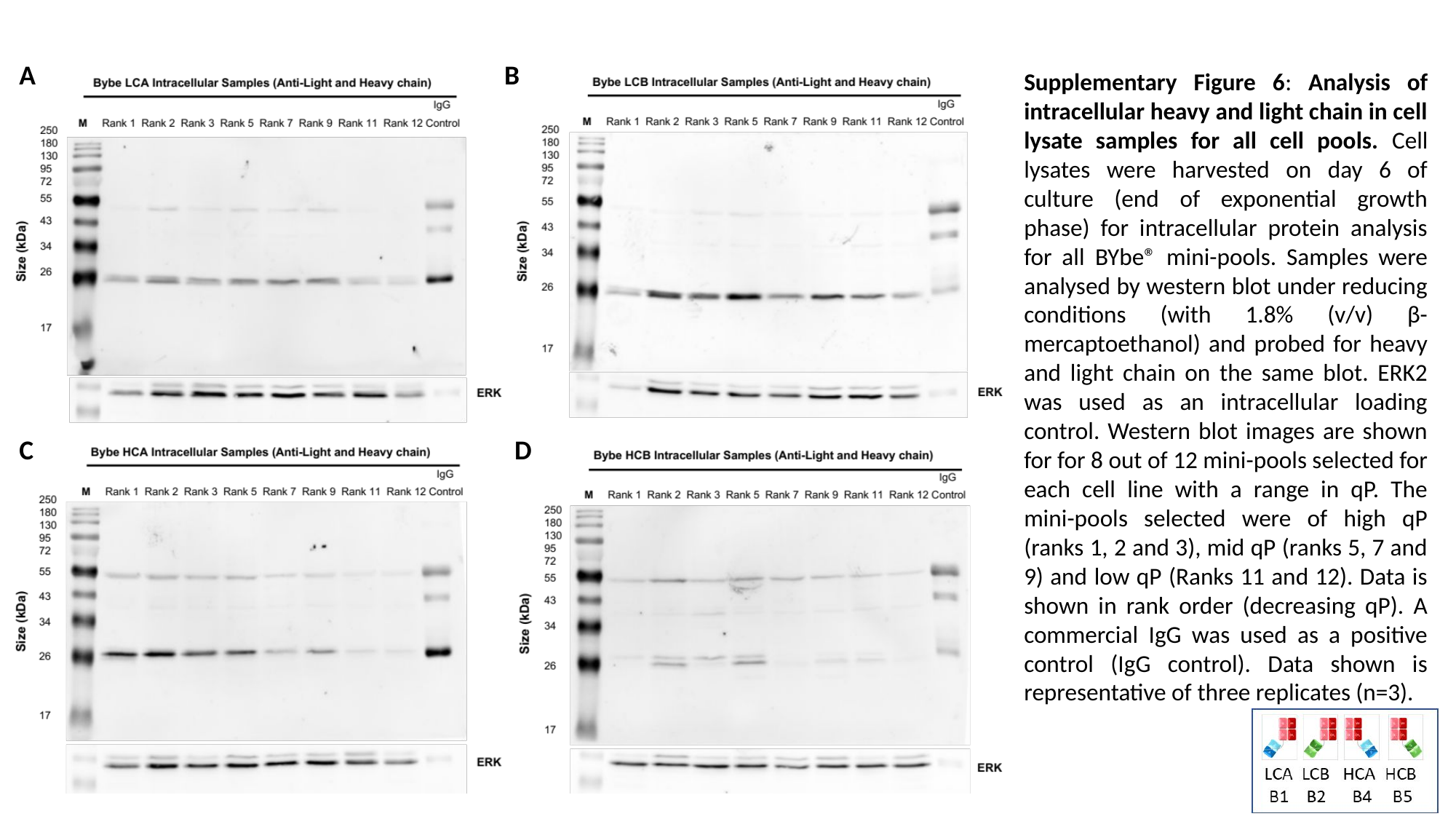

A B
C D
Supplementary Figure 6: Analysis of intracellular heavy and light chain in cell lysate samples for all cell pools. Cell lysates were harvested on day 6 of culture (end of exponential growth phase) for intracellular protein analysis for all BYbe® mini-pools. Samples were analysed by western blot under reducing conditions (with 1.8% (v/v) β-mercaptoethanol) and probed for heavy and light chain on the same blot. ERK2 was used as an intracellular loading control. Western blot images are shown for for 8 out of 12 mini-pools selected for each cell line with a range in qP. The mini-pools selected were of high qP (ranks 1, 2 and 3), mid qP (ranks 5, 7 and 9) and low qP (Ranks 11 and 12). Data is shown in rank order (decreasing qP). A commercial IgG was used as a positive control (IgG control). Data shown is representative of three replicates (n=3).

## Slide 8
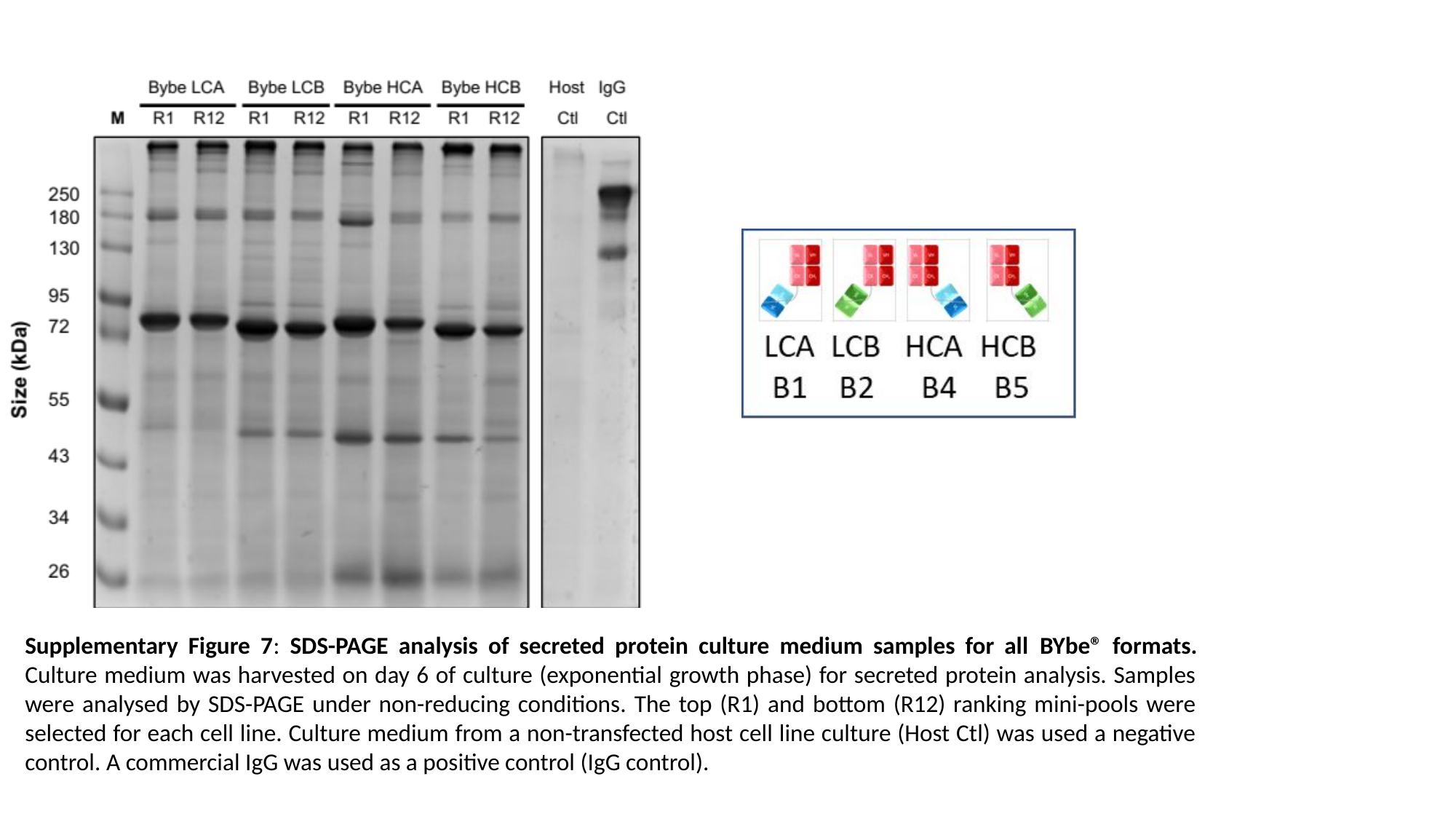

Supplementary Figure 7: SDS-PAGE analysis of secreted protein culture medium samples for all BYbe® formats. Culture medium was harvested on day 6 of culture (exponential growth phase) for secreted protein analysis. Samples were analysed by SDS-PAGE under non-reducing conditions. The top (R1) and bottom (R12) ranking mini-pools were selected for each cell line. Culture medium from a non-transfected host cell line culture (Host Ctl) was used a negative control. A commercial IgG was used as a positive control (IgG control).

## Slide 9
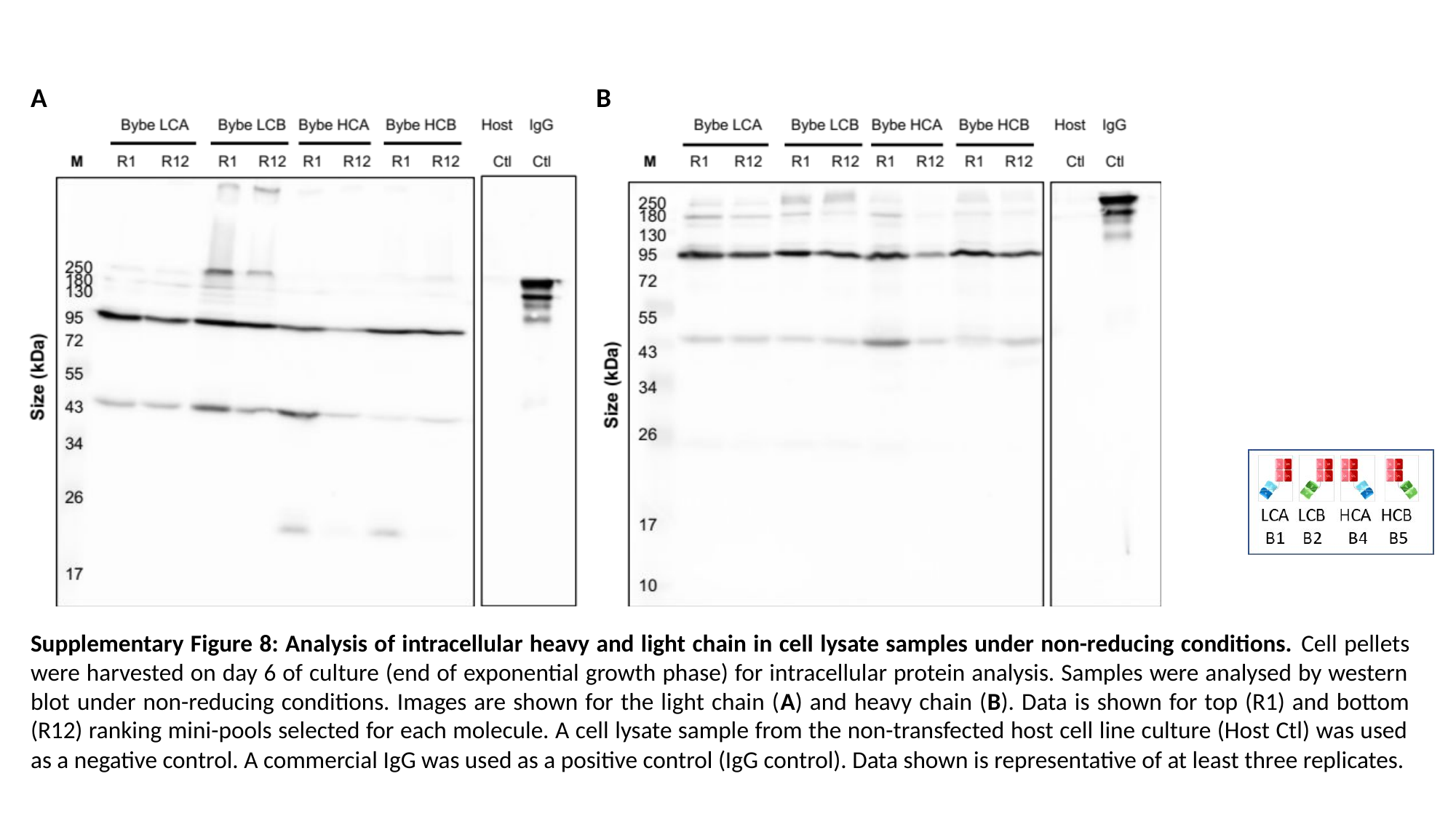

A B
Supplementary Figure 8: Analysis of intracellular heavy and light chain in cell lysate samples under non-reducing conditions. Cell pellets were harvested on day 6 of culture (end of exponential growth phase) for intracellular protein analysis. Samples were analysed by western blot under non-reducing conditions. Images are shown for the light chain (A) and heavy chain (B). Data is shown for top (R1) and bottom (R12) ranking mini-pools selected for each molecule. A cell lysate sample from the non-transfected host cell line culture (Host Ctl) was used as a negative control. A commercial IgG was used as a positive control (IgG control). Data shown is representative of at least three replicates.

## Slide 10
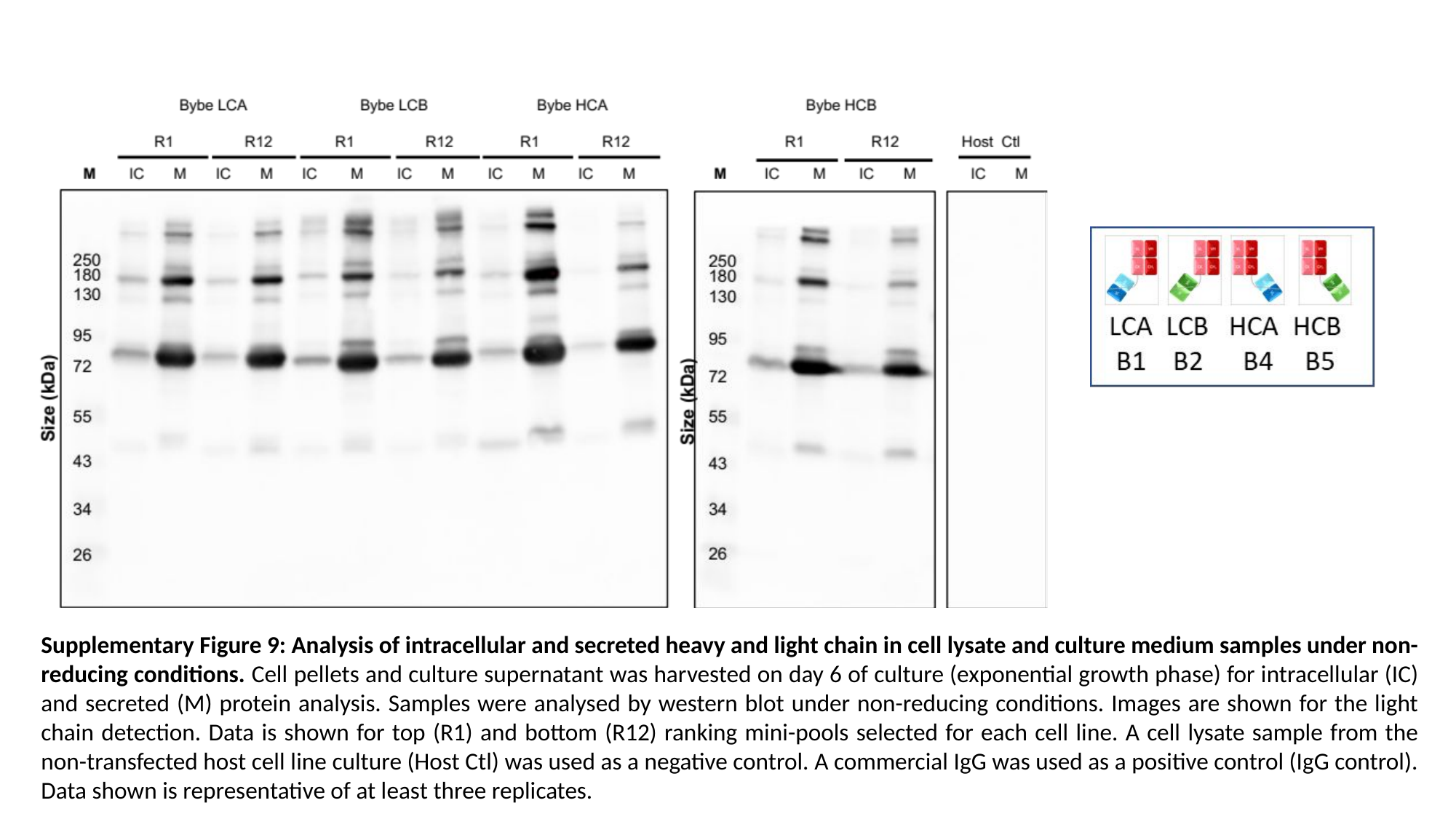

Supplementary Figure 9: Analysis of intracellular and secreted heavy and light chain in cell lysate and culture medium samples under non-reducing conditions. Cell pellets and culture supernatant was harvested on day 6 of culture (exponential growth phase) for intracellular (IC) and secreted (M) protein analysis. Samples were analysed by western blot under non-reducing conditions. Images are shown for the light chain detection. Data is shown for top (R1) and bottom (R12) ranking mini-pools selected for each cell line. A cell lysate sample from the non-transfected host cell line culture (Host Ctl) was used as a negative control. A commercial IgG was used as a positive control (IgG control). Data shown is representative of at least three replicates.

## Slide 11
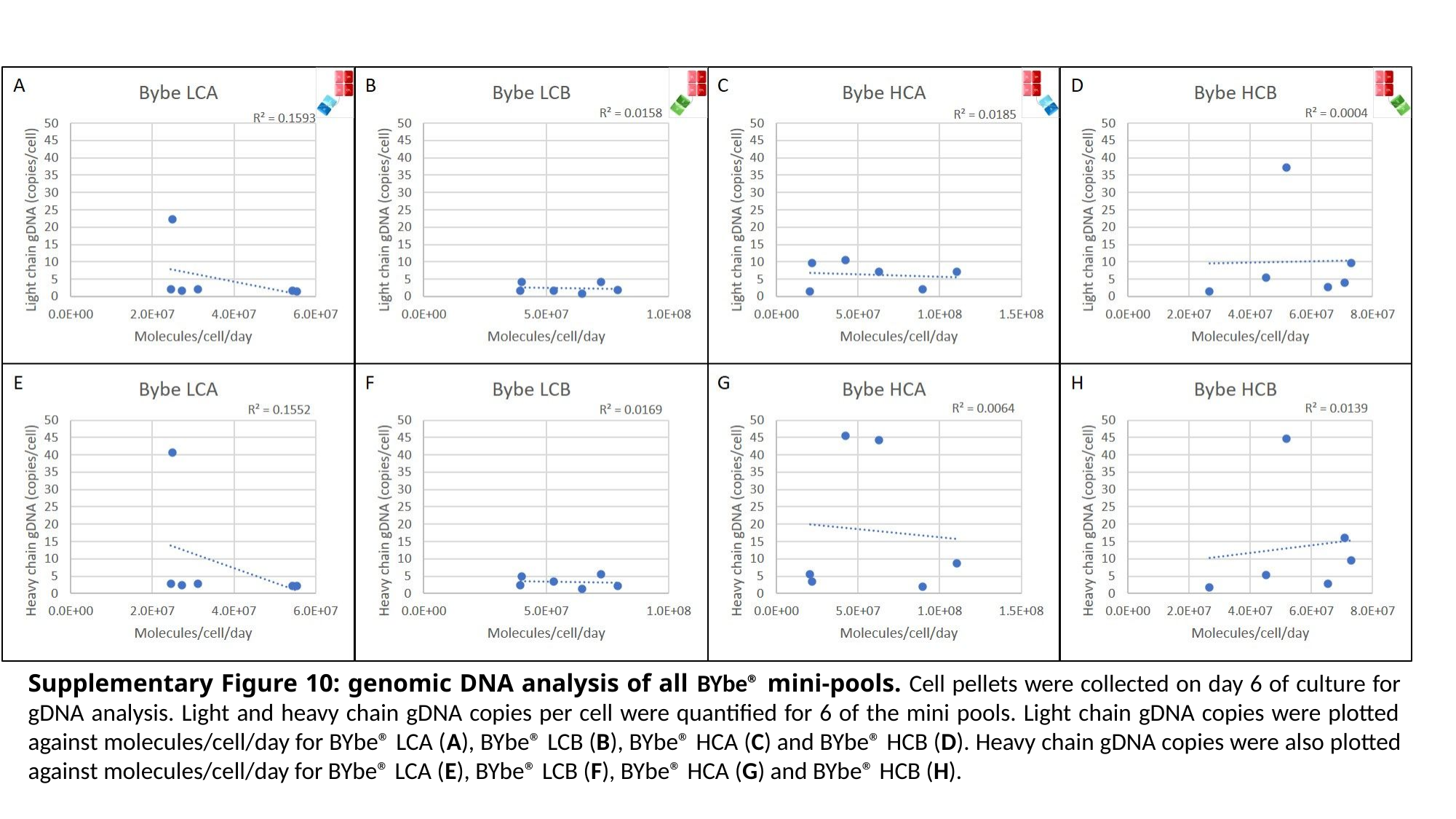

Supplementary Figure 10: genomic DNA analysis of all BYbe® mini-pools. Cell pellets were collected on day 6 of culture for gDNA analysis. Light and heavy chain gDNA copies per cell were quantified for 6 of the mini pools. Light chain gDNA copies were plotted against molecules/cell/day for BYbe® LCA (A), BYbe® LCB (B), BYbe® HCA (C) and BYbe® HCB (D). Heavy chain gDNA copies were also plotted against molecules/cell/day for BYbe® LCA (E), BYbe® LCB (F), BYbe® HCA (G) and BYbe® HCB (H).

## Slide 12
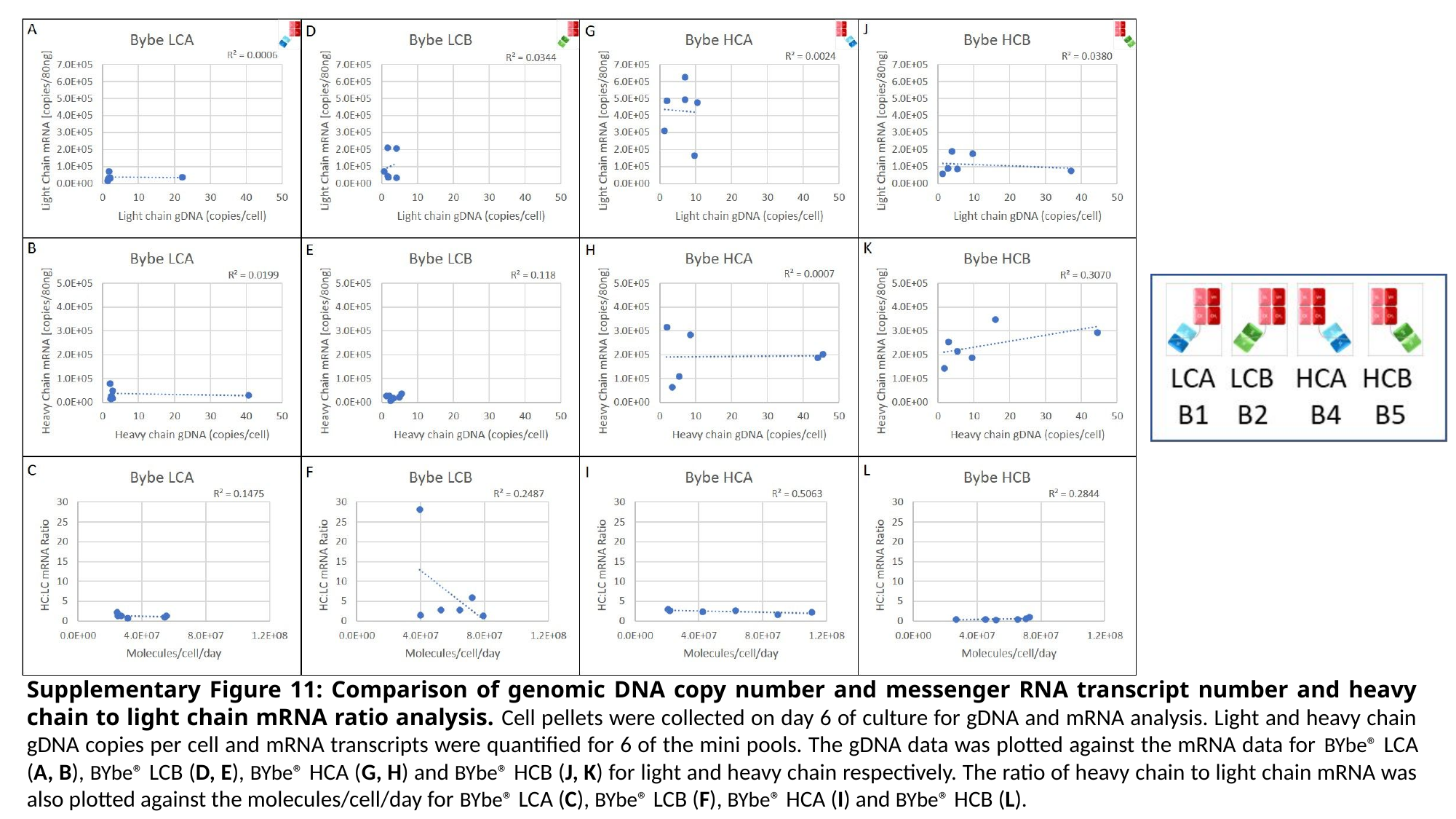

Supplementary Figure 11: Comparison of genomic DNA copy number and messenger RNA transcript number and heavy chain to light chain mRNA ratio analysis. Cell pellets were collected on day 6 of culture for gDNA and mRNA analysis. Light and heavy chain gDNA copies per cell and mRNA transcripts were quantified for 6 of the mini pools. The gDNA data was plotted against the mRNA data for BYbe® LCA (A, B), BYbe® LCB (D, E), BYbe® HCA (G, H) and BYbe® HCB (J, K) for light and heavy chain respectively. The ratio of heavy chain to light chain mRNA was also plotted against the molecules/cell/day for BYbe® LCA (C), BYbe® LCB (F), BYbe® HCA (I) and BYbe® HCB (L).
