# Supplementary material for: Sequence and Configuration of a Novel Bispecific Antibody Format Impacts Its Production Using Chinese Hamster Ovary (CHO) Cells
Source: Biotechnol Bioeng. 2024 Nov 25;122(2):435–44. doi: 10.1002/bit.28879 (PMC11718431; doi:10.1002/bit.28879)
Supplement: Supplementary file 2 — Supporting information. [file BIT-122-435-s002.docx]

**Supplementary Table 1**: Statistical analysis of data shown in Figure 2; productivity of top 12 BYbe® pools. The table summarises the statistical analysis of the plotted data in Figure 2 by one-way ANOVA, where P values of <0.05 (*), <0.01(**), <0.001(***) and <0.0001(****) were deemed significantly different.

|  | **Cell Productivity** | | |  | **Molecules/Cell/Day** | | |
| --- | --- | --- | --- | --- | --- | --- | --- |
| **Comparison** | **Statistically significant** | **P Value** | **Summary** |  | **Statistically significant** | **P Value** | **Summary** |
| Bybe LCA vs. Bybe LCB | Yes | <0.0001 | **** |  | Yes | <0.0001 | **** |
| Bybe LCA vs. Bybe HCA | Yes | <0.0001 | **** |  | Yes | <0.0001 | **** |
| Bybe LCA vs. Bybe HCB | Yes | <0.0001 | **** |  | Yes | <0.0001 | **** |
| Bybe LCA vs. hIgG1 X | Yes | <0.0001 | **** |  | Yes | <0.0001 | **** |
| Bybe LCA vs. hIgG1 Y | Yes | <0.0001 | **** |  | No | 0.2882 | ns |
| Bybe LCA vs. hIgG1 Z | Yes | <0.0001 | **** |  | No | 0.8840 | ns |
| Bybe LCB vs. Bybe HCA | No | 0.9990 | ns |  | No | 0.9911 | ns |
| Bybe LCB vs. Bybe HCB | No | >0.9999 | ns |  | No | 0.9995 | ns |
| Bybe LCB vs. hIgG1 X | Yes | <0.0001 | **** |  | No | 0.2239 | ns |
| Bybe LCB vs. hIgG1 Y | Yes | 0.0002 | *** |  | Yes | <0.0001 | **** |
| Bybe LCB vs. hIgG1 Z | No | >0.9999 | ns |  | Yes | <0.0001 | **** |
| Bybe HCA vs. Bybe HCB | No | >0.9999 | ns |  | No | >0.9999 | ns |
| Bybe HCA vs. hIgG1 X | Yes | <0.0001 | **** |  | No | 0.6430 | ns |
| Bybe HCA vs. hIgG1 Y | Yes | <0.0001 | **** |  | Yes | <0.0001 | **** |
| Bybe HCA vs. hIgG1 Z | No | 0.9972 | ns |  | Yes | <0.0001 | **** |
| Bybe HCB vs. hIgG1 X | Yes | <0.0001 | **** |  | No | 0.4550 | ns |
| Bybe HCB vs. hIgG1 Y | Yes | <0.0001 | **** |  | Yes | <0.0001 | **** |
| Bybe HCB vs. hIgG1 Z | No | 0.9997 | ns |  | Yes | <0.0001 | **** |
| hIgG1 X vs. hIgG1 Y | Yes | <0.0001 | **** |  | Yes | 0.0003 | *** |
| hIgG1 X vs. hIgG1 Z | Yes | <0.0001 | **** |  | Yes | <0.0001 | **** |
| hIgG1 Y vs. hIgG1 Z | Yes | 0.0003 | *** |  | Yes | 0.0157 | * |
